# Identifying Preferred Appearance and Functional Requirements of Aged Care Robots Among Older Chinese Immigrants: Cross-Sectional Study

**DOI:** 10.2196/48646

**Published:** 2023-11-08

**Authors:** Ching-Ju Chiu, Yi-Hsuan Lo, Jed Montayre, Hammoda Abu-Odah, Mei-Lan Chen, Ivy Yan Zhao

**Affiliations:** 1 Institute of Gerontology, College of Medicine, National Cheng Kung University, Tainan, Taiwan; 2 Department of Statistics, College of Management, National Cheng Kung University, Tainan, Taiwan; 3 World Health Organization Collaborating Centre for Community Health Services, School of Nursing, The Hong Kong Polytechnic University, Hong Kong, China; 4 School of Nursing, The Hong Kong Polytechnic University, Hong Kong, China; 5 School of Nursing, Byrdine F Lewis College of Nursing and Health Professions, Georgia State University, Atlanta, GA, United States

**Keywords:** robotic technology services, appearance, function, aged care, immigrant, Chinese, robot, robots, robotic, robotics, older adults, elderly, preference, cross sectional, cross-sectional, survey, healthy aging, aging in place, social, isolation, companion, companionship, Asian, Asian population, population, population studies, aging

## Abstract

**Background:**

Older Chinese immigrants constitute the largest older Asian ethnic population in New Zealand. Aging in a foreign land can be complex, presenting increasing challenges for gerontology scholars, practitioners, and policy makers. Older Chinese immigrants are more susceptible to experiencing loneliness and social isolation compared to native older people, primarily due to language, transportation, and cultural barriers. These factors subsequently impact their physical and mental health. With advancements in robotic technology, aged care robots are being applied to support older people with their daily living needs. However, studies on using robots with older immigrants living in the community are sparse. Their preferences for the appearance and function of aged care robots are unclear, which impacts the acceptance and usability of robots, highlighting the need for a user-centered design approach.

**Objective:**

This study aims to explore older Chinese immigrants’ needs and preferences toward the appearance and function of aged care robots and to examine their relationships with the demographic characteristics of participants.

**Methods:**

A cross-sectional design was used in this study, which was undertaken between March and May 2020. A total of 103 participants completed a web-based survey.

**Results:**

The average age of participants was 68.7 (SD 5.5) years. The results suggest that 41.7% (n=43) of the 103 participants preferred a humanlike adult appearance, while 32% (n=33) suggested an animallike appearance. These participants reported higher scores in both rigorousness and friendliness compared to others who preferred different robot appearances. Participants expressed a greater preference for the functions of housework assistance (n=86, 83.5%), language translation (n=79, 76.7%), health monitoring (n=78, 75.7%), facial expressions (n=77, 74.8%), news reading (n=66, 64.1%), and security monitoring (n=65, 63.1%). These preferences were found to be significantly associated with marital status, financial status, and duration of immigration.

**Conclusions:**

To support immigrant populations to age well in a foreign country and address the growing shortage of health and social professionals, it is important to develop reliable robotic technology services that are tailored based on the needs and preferences of individuals. We collected and compared the perspectives of immigrant and nonimmigrant participants on using robots to support aging in place. The results on users’ needs and preferences inform robotic technology services, indicating a need to prioritize older Chinese immigrants’ preference toward aged care robots that perform housework assistance, language translation, and health and safety monitoring, and robots with humanlike features.

## Introduction

Older Chinese immigrants are more prone to experiencing loneliness and social isolation than native older people due to language, transportation, and cultural barriers, which subsequently impact their physical and mental health [1]. In 2017, 11 million people emigrated from China to their destination countries [2]. New Zealand is one of the most popular host countries for Chinese immigrants, and as a result, it faces the growing needs of an increasingly diverse aging population. According to the New Zealand Census results in 2018, there were 247,770 Chinese immigrants 45 years or older, with 23,625 (9.5%) of them 65 years or older [3]. Compared to the statistics from the 2013 census, there was an increase of 76,359 (44.5%) Chinese immigrants [4]. Older Chinese immigrants are the largest older Asian ethnic population in New Zealand [3].

Aging in a foreign land can be complex, posing increasing challenges for gerontology scholars, practitioners, and policy makers. Self-supported aging in place has been reported as a benefit for enhancing older people’s health and quality of life, as it supports the continuity of the environment and promotes independent living within the community [5]. However, a large and rapidly growing social and health workforce shortage in New Zealand has been unable to meet the increasing needs of older Chinese immigrants to access social and care services in the community [6]. With innovations and advancements in computer systems, robotic technology and information and communication technologies have been applied to support older people with their daily living needs.

In a New Zealand study, a daily care robot was used at home to assist older community-dwelling adults who had different aging-related health needs. The robot’s purpose was to remind them of daily activities, and it showed promising potential in old age care, especially in providing reminders for taking medication [5]. Most existing studies on using robots to support older people have focused on dementia care and cognitive training, and have been undertaken in dementia care units. For example, a recent study in Italy reported that a humanoid robot called NAO effectively supported memory training among 24 patients with mild cognitive impairment, enhancing their therapeutic compliance and reducing symptoms of depression [7]. The study reported significant changes in prose memory and verbal fluency measures [7]. Additionally, the social robot Paro (an animal seal robot) was tested in Taiwan with 20 older adults in a long-term care facility for 8 weeks using a single-group pre-post quasi-experimental design and showed a statistical decrease in depression and loneliness and an increase in quality of life among the participants [8]. Our pilot study in Hong Kong has reported good feasibility and acceptance of using a humanoid social robot called KaKa among older Chinese adults and their family caregivers in their homes [9]. However, studies of using robots among older immigrants living in the community are sparse. Their preferences for the appearance and function of aged care robots are unclear, which impact the acceptance and usability of robots, and therefore, a user-centered design is required. It is imperative to understand users’ needs and preferences before designing and developing a robot to meet their specific care needs [10].

Aged care robots, including health care assistive robots and socially assistive robots, should be tailored for older people to be easy to use, flexible, and able to support natural older people–robot interaction [10]. In particular, the design should consider those people with less experience in using technological devices [10]. Gaseiger and colleagues [5] reported that older people living alone at home accepted a robot as a companion, and the functions of an aged care robot should be more personalized to meet older people’s health and social needs. A cross-sectional survey among middle-aged and older Taiwanese living in the community revealed that female participants preferred an animallike robot, while male participants favored a humanlike robot [11]. The most popular functions of a robot recommended by those participants included dancing, singing, storytelling, and news reading [11]. Moreover, the New Zealand study indicated that older Chinese immigrants were more likely to accept the companionship of robots when they were feeling lonely, yet more evidence is needed regarding their preferences for robot features [12]. Therefore, this study aimed to explore older Chinese immigrants’ needs and preferences toward the appearance and function of aged care robots. Additionally, it sought to examine the relationships between these needs and preferences and the demographic characteristics of participants.

## Methods

### Participants

Adults 60 years or older, as defined by the World Health Organization [13], who self-identified their ethnicity as Chinese; held a permanent resident visa in New Zealand or were New Zealand citizens; were able to read, write, and understand traditional or simplified Chinese; were able to access the internet; and had completed the web-based survey were eligible to participate. There were no specific exclusion criteria. We screened each participant’s eligibility through their individual demographic information.

### Instrument

**Figure 1.**
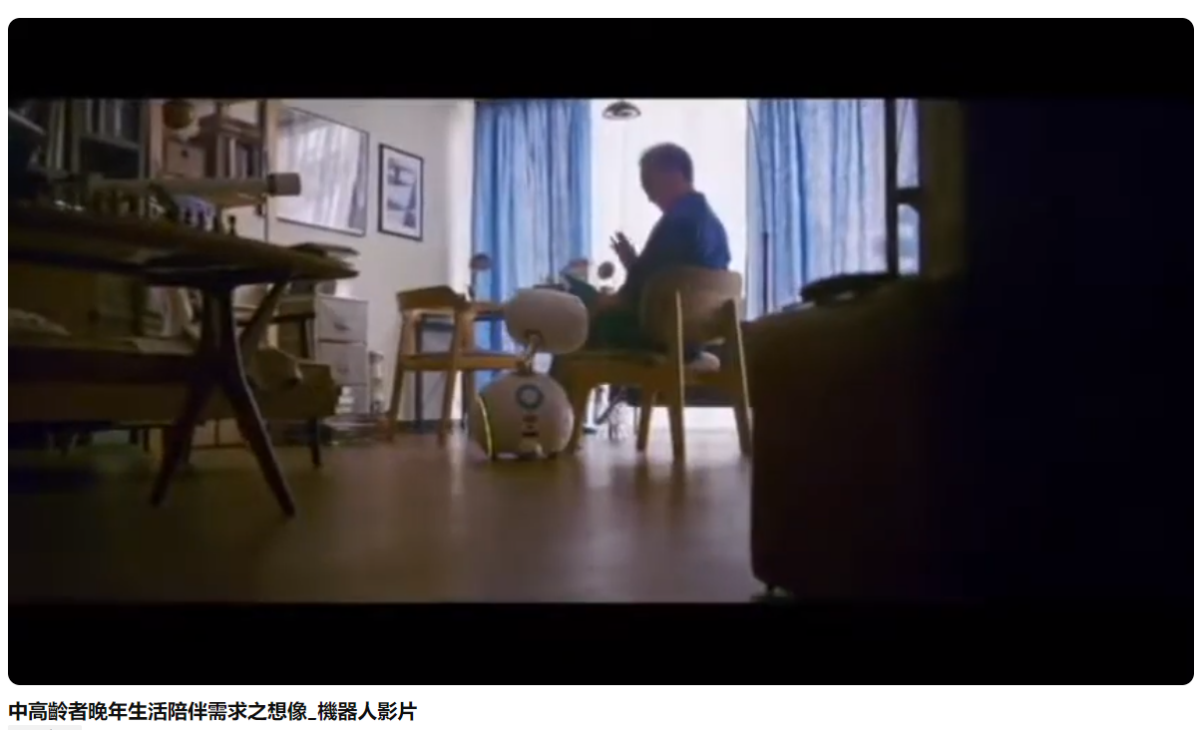
Screenshot of a video depicting an older adult chatting with a robot.

**Figure 2.**
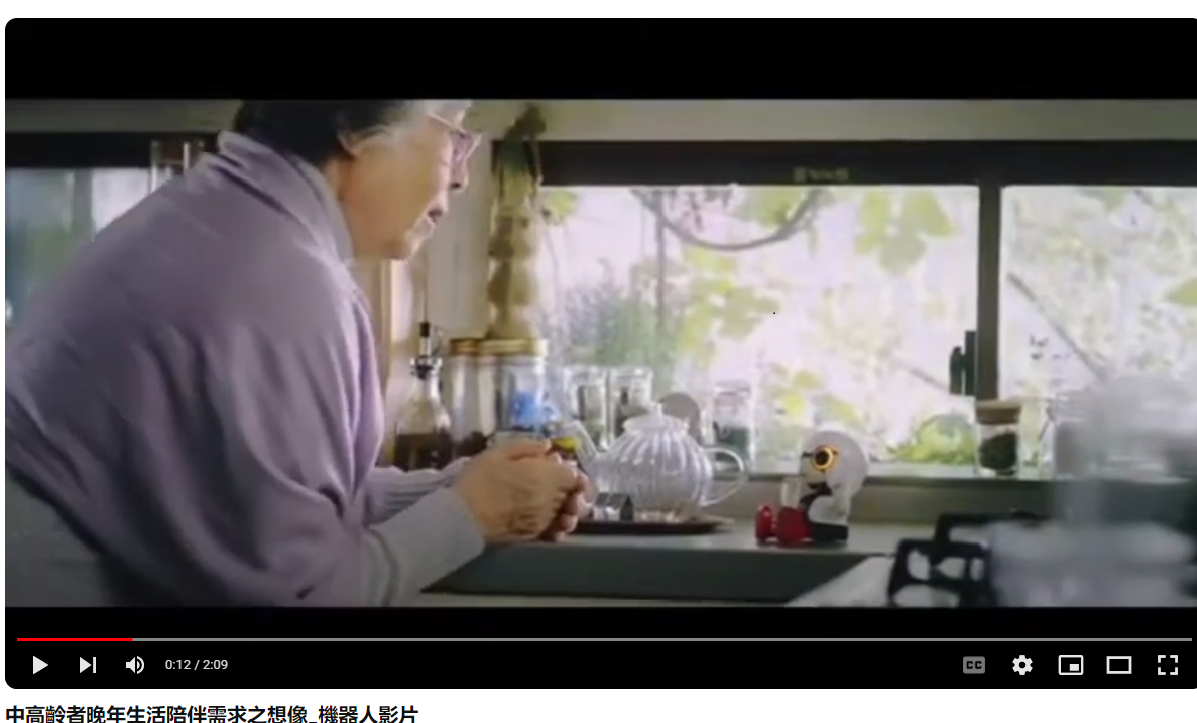
Screenshot of a video depicting a robot’s companionship role for an older lonely adult.

**Figure 3.**
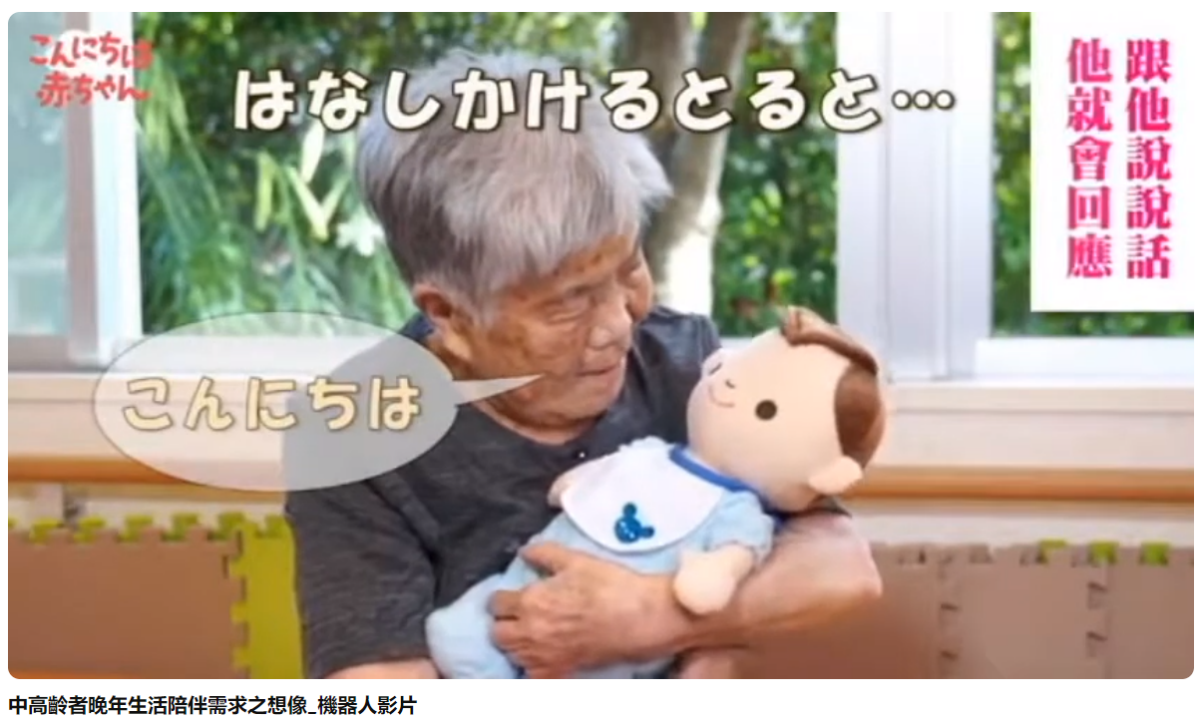
Screenshot of a video depicting a humanlike infant robot.

We used a web-based survey design tool, SurveyCake*,* to create a structured anonymous survey written in the traditional Chinese language. A simplified Chinese version was also made available as an alternative option. At the beginning of the survey, there was a 5-minute video using images from various online sources that was made for research purposes only. The video introduced different types of aged care robots, including health care assistive robots and socially assistive robots, each with a variety of appearances and features. The content of the video was presented in Mandarin with traditional Chinese subtitles, providing participants with a general idea about the types of robots and their capabilities. Following the video, participants were asked to complete the survey, which included four sections: (1) demographic information, (2) five personality traits, (3) eHealth literacy, and (4) preference for robot appearance and functions. Screenshots of the video are shown in Figures 1-3.

Participants were asked 18 questions regarding demographic information, including age, gender, education level, etc. The 15-item International Personality Item Pool, Five Personality Scale (extroversion, friendliness, rigorousness, emotional stability, and intelligence/imagination) was used [14]. The eHealth literacy of participants (ie, the internet use and search skills, ability to find reliable web-based content, and confidence in their abilities to search the internet) was assessed by the eHealth Literacy Scale [15]. Participants’ preferred or favorite appearance (ie, animallike, humanlike infant, humanlike adult, or another form) and functions of the robots (eg, assisting with housework, health monitoring, and instant language translation) were collected. The survey was developed based on our previous study in Taiwan on middle-aged and older Chinese adults [11].

### Ethical Considerations

The study protocol was approved by the institutional review board of the affiliated university (No. A-ER-105-509). Before participating in the web-based survey, each participant had to provide informed consent. They were introduced to the aims and content of the study, potential risks and benefits, and the right to withdraw from the study at any time. Each participant was asked to click a box to confirm their willingness to proceed. All respondents in this study have ticked this box, and their responses were anonymous.

### Procedure

The study was undertaken between March and May 2020 using a cross-sectional design. The recruitment was supported by a local social service organization for older people in Auckland and several Chinese community groups from different regions of Auckland. Following the ethics approval, an electronic version of the flyer (in both traditional and simplified Chinese), advertising the study and linking to the web-based survey, was circulated by social workers and community group leaders via word of mouth, WeChat groups, and WhatsApp groups. No incentives were offered for participation in this web-based survey study. The completeness check was done by two team members after the questionnaire had been submitted.

### Data Analysis

Descriptive analyses and inferential statistics were performed on R (version 4.1.1; R Foundation for Statistical Computing). Descriptive statistics were used for analyzing the demographic information, five personalities, eHealth literacy, and preference for robot appearance and functions. For each item, we calculated descriptive statistics as appropriate (eg, mean and SD or frequency and percentage). ANOVA and *χ*^2^ test were adopted to analyze the correlations between demographic factors and preferences for robot functions.

## Results

### Demographic Information

A total of 103 older Chinese immigrants completed the survey, resulting in a response rate of 89.6% (103/115). Among the participants, the minimum age was 60 years, and the maximum age was 87 years, with an average age of 68.7 (SD 5.5) years. Of the participants, 74 were female and 29 were male. Most of the 103 participants attained a bachelor’s degree or above (n=72, 70%), and had a good self-reported financial status (mean 22.7, SD 4.7). Most of the participants were married or had a partner (n=88, 85.4%) and lived with family or others (n=90, 87.4%). Most of them immigrated to New Zealand to reunite with family (n=83, 80.6%), and about half of them had lived in this country for more than 10 years. Participants reported higher scores in rigorousness and friendliness than other personalities. Details on the data distribution are shown in Table 1.

**Table 1.** Demographic information of participants (N=103).

Demographic variables		Values
Age (years), mean (SD)		68.7 (5.5)
**Gender, n (%)**		
	Female	74 (71.8)
	Male	29 (28.2)
**Level of education, n (%)**		
	Under bachelor’s degree	31 (30.1)
	Bachelor’s degree or above	72 (69.9)
**Marital status, n (%)**		
	Married or have a partner	88 (85.4)
	Unmarried, widowed, or no partner	15 (14.6)
**Live alone, n (%)**		
	Yes	13 (12.6)
	No	90 (87.4)
**Type of occupation, n (%)**		
	Nontechnical	15 (14.6)
	Semitechnical/technical	29 (28.2)
	Professional/management	52 (50.5)
	Others	7 (6.8)
**Whether or not retired, n (%)**		
	Retired	95 (92.2)
	Employed	8 (7.8)
**Reasons for immigration^a^, n (%)**		
	Job opportunity	7 (6.8)
	Family reunion	83 (80.6)
	Retirement	18 (17.5)
**Duration of immigration (years), n (%)**		
	<10	51 (49.5)
	≥10	52 (50.5)
**Original living place, n (%)**		
	Mainland of China	74 (71.8)
	Taiwan	16 (15.5)
	Hong Kong and Macau	9 (8.7)
	Other Asian countries or regions	4 (3.9)
Self-rated financial status (range 1-5), mean (SD)		3.6 (0.9)
**Five personality scale (range 0-15), mean (SD)**		
	Extroversion	10.3 (2.1)
	Friendliness	11 (2.1)
	Rigorousness	11.1 (2.1)
	Emotional stability	10.5 (2.4)
	Intelligence/imagination	9.7 (1.8)
eHealth literacy (range 8-40), mean (SD)		22.7 (4.7)

a Participants could provide multiple answers to this question.

### Participants’ Preferences for Appearance of Aged Care Robots

Table 2 shows that most of the 103 participants, both female and male, preferred a humanlike adult appearance (n=43, 41.7%), and their second preference was an animallike appearance (n=33, 32%). The remaining participants reported their preferences for a humanlike infant appearance (n=20, 19.4%) and other forms (n=6, 5.8%). Participants who preferred humanlike adult or infant and animallike appearances reported higher scores in rigorousness and friendliness than other personalities. Participants who desired other appearances, rather than animal and humanlike appearances, were more likely to report high scores in extroversion, friendliness, rigorousness, and emotional stability as well as in eHealth literacy. Participants who were married or had a partner were more likely to choose humanlike adult and animallike appearances.

**Table 2.** Participants’ preference for the appearance of a robot (N=103).

		Preference for robot’s appearance^a^			
		Animallike	Humanlike infant	Humanlike adult	Other forms
Participants, n (%)		33 (32.0)	20 (19.4)	43 (41.7)	6 (5.8)
**Gender, n (%)**					
	Female (n=74)	25 (33.8)	17 (23.0)	28 (37.8)	4 (5.4)
	Male (n=29)	8 (27.6)	3 (10.3)	15 (51.7)	2 (6.9)
**Personality (range 0-15), mean (SD)**					
	Extroversion	10.5 (2.2)	10.8 (2.0)	9.9 (2.2)	12.3 (2.0)
	Friendliness	11.2 (2.4)	11.3 (1.7)	11.0 (2.0)	12.3 (2.0)
	Rigorousness	11.4 (2.1)	11.1 (1.9)	11.2 (2.1)	12.5 (1.9)
	Emotional stability	10.7 (2.6)	10.9 (2.5)	10.4 (2.2)	12.3 (2.4)
	Intelligence/imagination	10.1 (2.0)	9.6 (1.5)	9.9 (1.7)	9.8 (1.5)
eHealth literacy (range 8-40), mean (SD)		23.6 (3.1)	21.7 (4.1)	23.6 (4.3)	25.3 (3.3)
**Marital status, n (%)**					
	Married or have a partner (n=88)	32 (36.4)	17 (19.3)	40 (45.5)	6 (6.8)
	Unmarried, widowed, or no partner (n=15)	1 (6.7)	3 (20.0)	3 (20.0)	0 (0.0)

a Participants could provide multiple answers to this question.

### Participants’ Most Preferred Functions of Aged Care Robots

Participants’ most preferred functions of aged care robots were housework assistance, language translation, health monitoring, facial expressions, news reading, and security monitoring. By analyzing the correlations between the demographic factors and the preferred functions of a robot, participants who were married or had a partner were more likely to choose functions of facial expressions (71/88, 81%; *P*=.002), news reading (61/88, 69%; *P*=.02), and security monitoring (60/88, 68%; *P*=.02) than their counterparts. High financial status showed a significant correlation with the function of facial expressions (mean 3.7, SD 0.7; *P*=.001). Participants who had immigrated for fewer than 10 years were more likely to use the function of facial expressions (43/51, 84%; *P*=.05) than people who had immigrated for 10 years or more. Detailed information is shown in Table 3.

**Table 3.** Participants’ most preferred six functions of robots (N=103).^a^

		Preference for robot functions^b^					
		Housework assistance	Language translation	Health monitor	Facial expressions	News reading	Security monitor
Participants, n (%)		86 (83.5)	79 (76.7)	78 (75.7)	77 (74.8)	66 (64.1)	65 (63.1)
**Marital status, n (%)**							
	Married or have partner (n=88)	72 (81.8)	70 (79.5)	69 (78.4)	71 (80.7)	61 (69.3)	60 (68.2)
	Unmarried, widowed, or no partner (n=15)	14 (93.3)	9 (60.0)	9 (60.0)	6 (40.0)	5 (33.3)	5 (33.3)
	*P* value	.46	.19	.23	*.002*	*.02*	*.02*
**Financial status**							
	Mean (SD)	3.6 (0.9)	3.6 (0.8)	3.6 (0.8)	3.7 (0.7)	3.6 (0.8)	3.6 (0.8)
	*P* value	.86	.49	.41	*.001*	.50	.42
**Duration of immigration (years), n (%)**							
	<10 (n=51)	41 (80.4)	41 (80.4)	41 (80.4)	43 (84.3)	34 (66.7)	32 (62.7)
	≥10 (n=52)	45 (86.5)	38 (73.1)	37 (71.2)	34 (65.4)	32 (61.5)	33 (63.5)
	*P* value	.57	.52	.39	.05	.74	>.99

a Statistically significant results (*P*<.05) are in italics.

b Participants could provide multiple answers to this question.

## Discussion

### Principal Findings

With the advancement of robotic technology, care robots are being used to assist older adults with their daily living needs. However, there is a lack of research on the use of robots with older immigrants residing in the community and their preferences regarding the appearance and functionality of aged care robots. Therefore, this study aimed to investigate the needs and preferences of older Chinese immigrants toward the appearance and function of aged care robots, and examine the relationship between these preferences and the demographic characteristics of the participants. The findings revealed that older Chinese immigrants favored a humanlike adult appearance for the robots. Additionally, participants with different marital status, financial status, and duration of immigration had varying needs for the robot’s functionalities.

### Robot Appearance

In this study, the most popular appearance of a robot rated by older Chinese immigrants were those with a humanlike adult appearance, where no difference was identified between female and male participants. Our findings are consistent with Chiu et al’s [11] study that the favorite appearance of robots among middle-aged and older Taiwanese people was a humanlike adult appearance, but the correlation to marital status was less significant. Prakash and Rogers [16] reported distinctive differences in preferences for a robot appearance between young and older adults, whereas older adults had a higher preference for humanlike robots. The authors suggested that the differences might be attributed to their experiences with robots [16], and older adults felt comfortable talking with robots with human traits such as eyes and a mouth [17]. Moreover, a rural study in China reported that older adults perceived small-sized robots as more friendly, and steel machinelike robots were less preferred [18].

According to the research on human-robot interaction, the appearance and morphology of a robot are known to be important in increasing the acceptance and use of and interaction with a robot among older adults [19]. However, there is less evidence showing that robots were developed based on the characteristics of older adults. This study uniquely found that older Chinese immigrants who preferred humanlike adult or infant appearances reported higher scores in rigorousness and friendliness. This knowledge may help inform future robot designs for older adults from a morphological perspective [19].

### User Needs and Preferences

Results of the study showed that participants with different marital status, financial status, and duration of immigration had different needs for robot functions. Designing robot services to support older people must be based on individually collected information [20]. In this study, older Chinese immigrants mostly desired family service functions (ie, housework assistance), language translation, health monitoring, facial expressions, news reading, and security monitoring. The findings were different from the reported results among middle-aged and older Taiwanese, where the most preferred functions were the skill and recreation functions, followed by family services (ie, housework) and then health status monitoring [11]. The difference might be explained by the immigrant context of individual circumstances and the purpose of immigration. In this study, over 80% of the participants immigrated to reunite with their adult children, which is aligned with Zhao et al’s [21] study that found that most Chinese late-life immigrants relocated to New Zealand to share house chores with their adult children or look after their grandchildren. The burden of housework adversely impacted their health and became a risk factor for their experiences of loneliness and social isolation [1,12,21]. The same issue was also observed among other Asian immigrant groups due to the value of filial piety [22]. Assistive functions of robots were required by participants to relieve their workload and address the language barrier in a host country.

The evidence of this study found that the safety- and health-monitoring functions of the robot were regarded as essential for participants to meet their health and well-being needs, and our data supports Chiu et al’s [11] findings that different ages were significantly related to the preference for the safety-monitoring function of the robot. Most of our participants lived independently and expected to maintain their independence, which is consistent with Park et al’s [20] study that early detection of emergencies by using robot technology to assist with community-dwelling older adults’ daily living is necessary. Moreover, living with others was significantly correlated to participants’ preference for the health-monitoring robot function, which might be justified by several studies in New Zealand on Asian immigrant groups that have barriers to access health services due to their language, culture, and transportation barriers, and they intended to stay healthy and avoid becoming a burden to their family [1,21,23].

Moreover, participants who were married or had a partner, had good financial status, and had immigrated fewer than 10 years ago were more likely to choose the function of facial expressions. The finding is consistent with the previous study that older adults with lower technology acceptance preferred friendly and familiar robot designs with humanlike facial features [19]. However, the needs of those who were single with lower education levels might be underreported in this study, as the majority of our participants had higher education levels and were married or had a partner.

### Limitations and Future Work

This study recognizes several limitations. First, due to the web-based survey, we might have excluded potential participants without access to the internet, computer, cellphone, etc, or who were not able to answer the survey on the web. Second, it is possible that the responses of participants were biased due to the self-reported data. Third, the data collection was undertaken in Auckland. The generalizability of the study results for the whole of New Zealand and other destination countries is limited. In the future, larger representative samples are needed to further investigate the needs and preferences of using robots in the later phase of life and to generalize the relationships between demographic factors, characteristics of older adults, and preferences for robots among immigrant populations. Mixed methods and co-design research methods are recommended to gain in-depth insights into end users’ needs and preferences for robots to support their functions and independence in old age.

### Conclusion

To support immigrant populations to age well in a foreign country and to fill the gaps of increasing shortages in the health and social workforce, it is important to develop reliable robotic technology services that are tailored based on the needs and preferences of individuals. We collected and compared the opinions on using robots to support aging in place among immigrant and nonimmigrant groups. The results of users’ needs and preferences would inform robotic technology services to prioritize older Chinese immigrants’ preference toward housework assistance, language translation, health and safety monitoring, and robots with humanlike features.
